# Vertebral column resection and homogeneous spinal-shortening axial decompression for tethered cord syndrome: a meta-analysis and meta regression

**DOI:** 10.3389/fsurg.2025.1676474

**Published:** 2025-12-05

**Authors:** Dewa P. W. Wardhana, Robert E. Djojoseputro, Angela F. Ciaves, Jane C. Sutedja, Bryan G. D. Liyis

**Affiliations:** 1 Departement of Neurosurgery, Medical Faculty of Udayana University, Udayana University Hospital Denpasar, Bali, Indonesia; 2 Department of Neurosurgery, Doctor Soekardjo State Regional Hospital, Tasikmalaya, West Java, Indonesia; 3 Faculty of Medicine, Universitas Udayana, Denpasar, Bali, Indonesia; 4 Department of Neurosurgery, National Brain Center Hospital Mahar Mardjono, Jakarta, Indonesia

**Keywords:** homogeneous spinal-shortening axial decompression, vertebral column resection, tethered cord syndrome, spinal column shortening, neurological outcomes, complications

## Abstract

**Introduction:**

Tethered cord syndrome (TCS) is a group of neurological symptoms due to increased tension along the spinal cord. Surgical untethering has been the conventional treatment to relieve the spinal cord tension. However, untethering is associated with retethering of the spinal cord and the concerns regarding intradural nerve components manipulation. Recently, spinal column shortening (SCS) is emerging as an alternative treatment. This study aims to compare the efficacy of vertebral column resection (VCR) and homogeneous spinal-shortening axial decompression (HSAD) in improving neurological outcomes in TCS and the associated complications.

**Methods:**

A PROSPERO-registered systematic search was conducted in the ScienceDirect, PubMed, Embase, and Cochrane databases to identify longitudinal studies up to August 2024 (CRD42024566861). SCS techniques assessed includes VCR and HSAD. Data were extracted on patient demographics, clinical improvements, and complication rates. Single-arm outcomes were pooled using a random-effects GLMM with a logit link. Heterogeneity was assessed and potential moderators were examined through meta-regression. Freeman-Tukey method was used for sensitivity analyses. Publication bias was assessed using Egger's regression test and trim-and-fill analysis. All analyses were conducted with STATA software (*p* < 0.05).

**Results:**

The meta-analysis included 15 studies comprising 251 participants (44.2% male; mean age 28.37 ± 5.7 years), with mean operation time of 309.98 ± 53.35 min, estimated blood loss of 1,074.36 ± 338.51 mL, and follow-up duration of 36.95 ± 6.83 months. HSAD had shorter operation time (*p* = 0.01) and lower EBL (*p* = 0.04) compared to VCR. Both techniques yielded great improvement in pain, motor and sensory function, bowel and bladder function. HSAD produced significantly greater bladder function improvement compared to VCR (82% vs. 51%, *p* < 0.001). Complication rate was lower in HSAD compared to VCR (7% vs. 17%), with borderline statistical significance (*p* = 0.05). Meta regression analyses supported the statistical robustness, especially for pain and bladder function improvement with a constant *p*-value of 0.03 and 0.02, respectively.

**Conclusions:**

VCR and HSAD both offer significant neurological improvements and satisfactory patient outcomes with no comparable significant complications. HSAD has the greater potential to improve bladder function in TCS. Patient treatment selection should be individualized depending on the presence of comorbidity and surgeon's experience.

**Systematic Review Registration:**

https://www.crd.york.ac.uk/PROSPERO/view/CRD42024566861, identifier CRD42024566861.

## Introduction

Tethered cord syndrome (TCS) is a group of clinical complaints caused by increased tension along the spinal cord. The increased tension caused by an abnormal attachment of spinal cord to immobile points. Children are more commonly affected by TCS compared to adult. TCS is estimated to be found in 1 per 4.000 live births. Clinical manifestations vary from pain, motor/sensory abnormality, bowel/bladder dysfunction. Magnetic resonance imaging (MRI) is currently the gold standard of diagnosis and can help in surgical decision making (1, 2). Treatment principle is relieving the spinal cord tension which, traditionally, can be achieved by surgical untethering. However, untethering can be challenging in complex cases, such as presence of tumors or post-operative scarring, where attachments can be difficult to find (3). Traditional untethering surgery also requires opening of the thecal sac and involves manipulation of the nerve components. The risk of cerebrospinal fluid (CSF) leak is also worth mentioning. Studies are also reporting post-operative fibrosis leading to formation of spinal cord tethering (4, 5).

Spinal column shortening (SCS) techniques are being considered as an alternative option for complex TCS (6). In contrast to untethering, SCS procedures avoid opening of the thecal sac and manipulation of the neural components. Risk of CSF leakage is also relatively minimal with SCS (7, 8). Studies are reporting promising outcomes from vertebral column resection (VCR) and homogeneous spinal-shortening axial decompression (HSAD) as an alternative or revision surgery in TCS (9–23). A comprehensive and solid analysis of the clinical effectiveness and safety of SCS in TCS patients is required, given the growing interest and evidence reported in recent years. This study aims to compare the clinical outcomes and complications associated with TCS patients undergoing VCR or HSAD.

## Methods

A PROSPERO-registered systematic search was conducted in the ScienceDirect, PubMed, Embase, and Cochrane databases to identify relevant longitudinal studies up to August 2024 (CRD42024566861). The search was conducted across databases, including PubMed, EMBASE, Cochrane, with keywords focused on patients with tethered cord syndrome who underwent spinal column shortening procedures. We included pediatric and adult patients, regardless of the presence of previous surgery for TCS. Clinical outcomes were chosen as follows: pain improvement, sensory and motor functions, bladder and bowel functions, and complications. Spinal column shortening techniques includes VCR and HSAD. Statistical analyses were performed using random-effects models with STATA software.

### Study design and inclusion criteria

This systematic review was conducted following the Preferred Reporting Items for Systematic Reviews and Meta-Analyses (PRISMA) guidelines to ensure transparency and methodological rigor (24). The review protocol was prospectively registered and approved with the International Prospective Register of Systematic Reviews (PROSPERO) under the registration number CRD42024566861 (25).

Inclusion criteria on this study were based on the PICO (Population, Intervention, Comparison, Outcome) format. The population was set to patients of any age diagnosed with radiographically confirmed TCS. VCR was chosen as the intervention, while HSAD was the comparator. Outcomes were pain relief, sensory and/or motor function improvements, bowel/bladder function, and complications. We also required the studies to report at least one of the following variables: estimated blood loss (EBL), operation time, and follow-up duration. As for the types of study, we included prospective and retrospective cohort studies, and case series. Our exclusion criteria were presence of any other spinal conditions or history of spinal surgeries unrelated to TCS. We also excluded studies with incomplete clinical outcomes. Studies with follow-up duration less than 12 months and reporting less than 3 patients were also excluded from our review.

### Literature search and selection

A comprehensive literature search was performed across multiple electronic databases, including ScienceDirect, PubMed, Embase, and Cochrane to identify relevant studies published up to August 2024. The search strategy utilized Medical Subject Headings (MeSH) terms related to “tethered cord syndrome,” “spinal column shortening,” “vertebral column resection,” and “homogeneous spinal-shortening axial decompression.” Table 1 describes the operational variables for PICO and also the search strategy used across the databases. No language restrictions were applied, and both pediatric and adult populations were considered. Manual assessments of the studies were then conducted to further examine and ensure the quality of the studies selected. Any relevant references to the subject were identified. Eventually, we gathered 15 studies in total, including 14 retrospective studies and 1 case series study (9–23).

**Table 1:** PICO and search strategy.

Element	Description
Population (P)	Patients of any age diagnosed with TCS, including primary or recurrent TCS, regardless of etiology
Intervention (I)	VCR, defined as posterior vertebral column resection osteotomy with the intention of reducing spinal column height to relieve spinal cord tension without direct spinal cord manipulation. Procedures include single-level osteotomy.
Comparator (C)	HSAD, defined operationally as SCS procedure performed via modified SSO. HSAD is performed by uniform limited osteotomy on multilevel, followed with application of slow and homogeneous shortening of the vertebral column aimed to relieve spinal cord tension without direct spinal cord manipulation.
Outcome (O)	Primary outcome:
	- Functional and neurological improvement, including pain, motor function, sensory function, bowel function, and bladder function. - Secondary outcome: - Intra-operative variables (EBL, operation time) and complications following the surgery
Study design (S)	Randomized controlled trials, prospective or retrospective cohort studies, and case series with >3 patients reporting postoperative outcomes for either VCR or HSAD procedures. Case reports, editorials, reviews, and animal studies were excluded.
Databases searched	ScienceDirect, PubMed, Embase, and Cochrane
Date of final search	August 9th, 2024 (all databases included studies from inception to August 2024)
Language/Publication type	English and Chinese studies were included. Conference abstracts and grey literature were excluded.
Search string used	
ScienceDirect	(“tethered cord syndrome” OR “tethered spinal cord” OR “recurrent tethered cord”)
	AND (“spine shortening” OR “spinal column shortening” OR “vertebral column shortening”
	OR “vertebral column resection” OR “posterior vertebral column resection”
	OR “spinal-shortening osteotomy” OR “axial decompression”
	OR “homogeneous spinal-shortening axial decompression”)
	AND (surgery OR surgical OR operation OR osteotomy OR outcome)
PubMed	(“Tethered Cord Syndrome”[Mesh] OR “tethered cord”[tiab] OR “tethered spinal cord”[tiab]
	OR “secondary tethered cord”[tiab] OR “recurrent tethered cord”[tiab]) AND (“spine shortening”[tiab] OR “spinal shortening”[tiab] OR “spinal column shortening”[tiab] OR “vertebral column shortening”[tiab] OR “spinal-shortening osteotomy”[tiab] OR “vertebral column resection”[tiab] OR “posterior vertebral column resection”[tiab]
	OR “vertebral column subtraction osteotomy”[tiab] OR “spinal column resection”[tiab] OR “axial decompression”[tiab] OR “homogeneous spinal-shortening axial decompression”[tiab])
	AND (surg*[tiab] OR operativ*[tiab] OR osteotomy[tiab] OR “clinical outcome”[tiab] OR “treatment outcome”[tiab])
	Filters: Humans, English
Embase	(“tethered cord syndrome”/exp OR “tethered cord”:ti,ab OR “recurrent tethered cord”:ti,ab
	OR “secondary tethered cord”)
	AND (“spine shortening”:ti,ab OR “spinal column shortening”:ti,ab OR “vertebral column shortening”:ti,ab OR “spinal-shortening osteotomy”:ti,ab OR “vertebral column resection”:ti,ab OR “posterior vertebral column resection”:ti,ab OR “vertebral column subtraction osteotomy”:ti,ab OR “axial decompression”:ti,ab OR “homogeneous spinal-shortening axial decompression”:ti,ab)
	AND (surg*:ti,ab OR operativ*:ti,ab OR osteotomy:ti,ab OR outcome*:ti,ab) AND [humans]/lim
Cochrane	(“tethered cord syndrome” OR “tethered spinal cord” OR “secondary tethered cord” OR “recurrent tethered cord”)
	AND (“spinal shortening” OR “spinal column shortening” OR “vertebral column resection” OR “posterior vertebral column resection” OR “vertebral column subtraction osteotomy” OR “spinal-shortening osteotomy” OR “axial decompression”
	OR “homogeneous spinal-shortening axial decompression”) AND (surg* OR osteotomy OR operation OR outcome)

EBL, estimated blood loss; HSAD, Homogeneous Spinal-shortening Axial Decompression; SCS, spinal column shortening; SSO, spinal shortening osteotomy; TCS, tethered cord syndrome; VCR, Vertebral Column Resection.

### Data extraction

The data extraction started with collecting information on the patient demographics, characteristics, clinical problems, intra-operative variables, and post-operative outcomes from the studies. The demographics collected included age sex; operative variables included type of SCS procedure (VCR or HSAD), estimated blood loss (EBL), and operation time; while clinical variables included pain score improvement, motor/sensory function improvement, bowel/bladder function, follow-up period, and post-operative complication. Data extraction was carried out independently by two reviewers using a standardized form. Any discrepancy between reviewers were discussed thoroughly and resolved accordingly.

### Quality assessment and data analysis

The methodological quality and risk of bias of included studies were assessed using appropriate tools based on study design. The Cochrane Risk of Bias in Non-Randomized Studies—of Interventions (ROBINS-I) tool was utilized as all the studies selected were non-randomized. The statistical methodology employed aimed at precision, representing continuous data as mean ± standard deviation and categorical variables as absolute numbers or percentages. Incorporating 95% confidence intervals (CIs) in the presentation enabled comparisons between studies.

Synthesis of single-arm outcomes was performed by pooling proportions using a random-effects generalized linear mixed model (GLMM) with a logit link and study-specific random intercept fitted to the binomial likelihood (events/total). This approach directly models within-study binomial variance, accommodates rare and zero- or all-event studies without continuity corrections, and yields model-based 95% Cis that perform well even near boundary values. Pooled legit estimates and Cis were back-transformed to the proportion scale for interpretation. Heterogeneity was quantified using τ^2^ (restricted maximum likelihood, REML), *I*^2^, and the Cochrane Q statistic. Where feasible, 95% prediction intervals were also reported. Study-level proportions are presented with exact Clopper-Pearson Cis.

Random-effects meta regression was conducted using the REML estimator to assess the potential influence of study-level covariates on effect size. The significance of each moderator was evaluated using the Wald chi square test and model fit was summarized with *R*^2^ and residual heterogeneity statistics. Included covariates were the proportion of prior untethering, male participants, mean age, mean EBL, and mean operation time.

As a sensitivity analysis, all proportion meta-analyses were repeated using the Freeman-Tukey double-arcsine (PFT) transformation under a random-effects model with REML estimation of *τ*^2^ and inverse-variance weighting. The PFT method, which also accommodates zero- and 100%-event studies, stabilizes sampling variation when proportions approach 0 or 1. Pooled estimates were converted to the proportion scale using Miller's method. The REML model was utilized to combine effect estimates, accounting for variations in treatment effects, while the Mantel-Haenszel method was employed to evaluate heterogeneity and guide model selection. Publication bias was assessed using Egger's regression test and trim-and-fill procedure. All analyses were carried out using STATA software version 18 (StataCorp, College Station, TX, USA), with statistical significance set at *p* ≤ 0.05.

## Results

### Study selection

**Figure 1:**
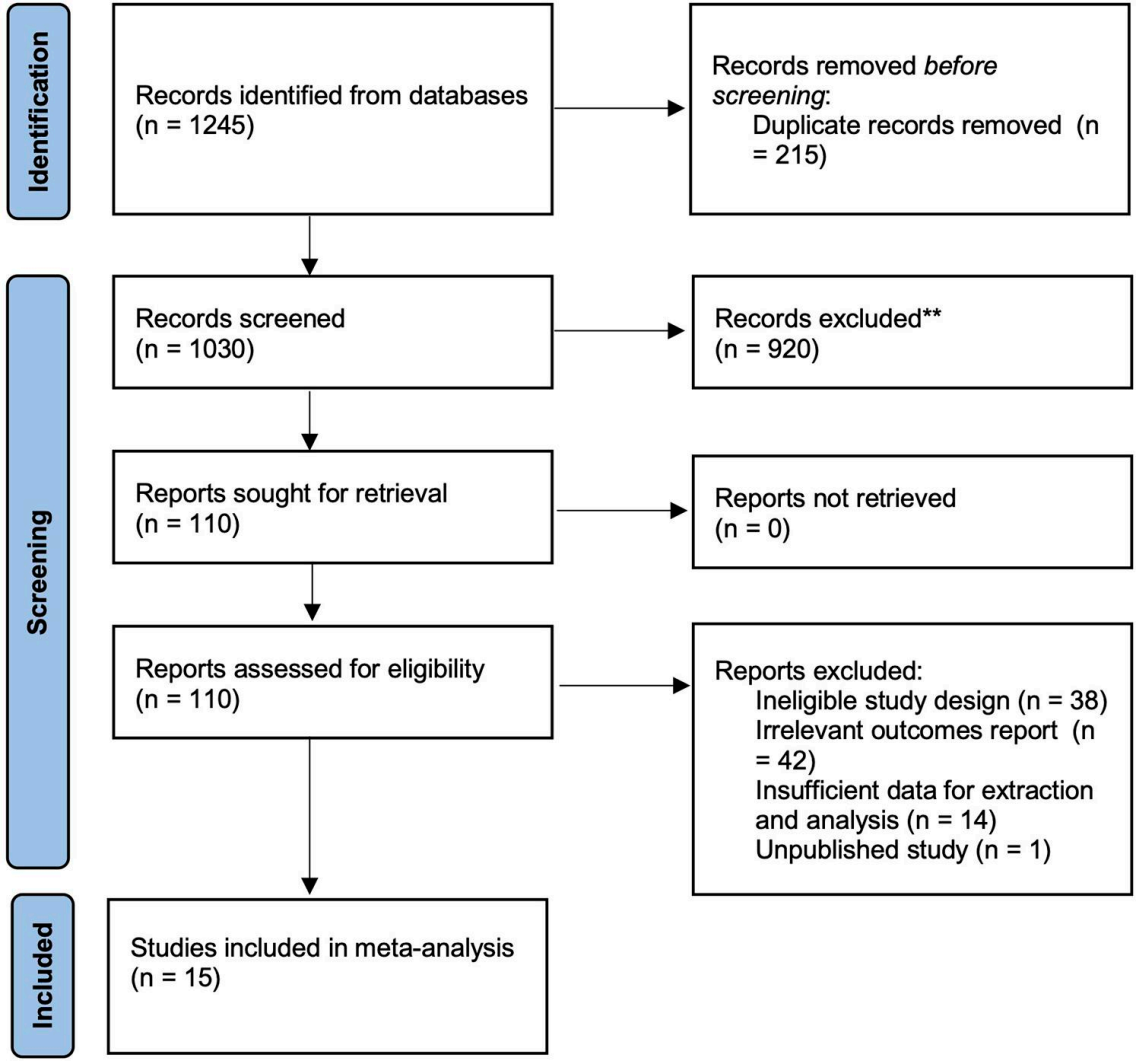
PRISMA flowchart.

The systematic search found 1,245 records with 215 duplicate records. After removing the duplicates, 1,030 records were then screened for relevance to the research question. A total of 920 records were excluded due to several reasons, such as irrelevant focus (not pertaining to TCS or SCS procedures), non-original research articles, and non-clinical studies. The full-texts of the remaining 110 articles were assessed for eligibility and we excluded 95 records due to unpublished study, ineligible study design (e.g., case reports, studies with fewer than 3 patients), irrelevant outcomes report, insufficient data for extraction or analysis. Finally, the remaining 15 studies were selected and included in the meta-analysis. There are 9 studies on VCR included and 6 studies on HSAD. Figure 1 shows the PRISMA diagram of the systematic review. The selected studies were then assessed for bias in non-randomized studies using the ROBINS-I tool.

### Study characteristics

This systematic review encompassed 15 studies involving a total of 251 patients who underwent spinal column shortening procedures, specifically VCR and HSAD, for treatment of TCS. Table 2 provides the demographic and characteristics of the included studies. The studies varied in design, including 1 retrospective case series (14) and 14 cohort studies with patient populations including both pediatric and adult groups (9–13, 15–23). The included studies originated from several different countries, 8 studies were from China (*n* = 172 total patients), 4 studies were from Japan (*n* = 21), and 3 studies were from USA (*n* = 58). The etiology of TCS was reported in all of the studies. The surgical techniques employed were VCR and HSAD, where there were 9 studies used VCR (*n* = 106) and 6 studies used HSAD (*n* = 145). Different patient population was reported in the studies, they were further grouped into adult only (*n* = 5), mixed (*n* = 9), and pediatric only (*n* = 1). Included studies were also grouped based on their mean follow-up period (less or more than 3 years) for further subgroup analysis. Operative variables were variedly reported in the studies, estimated blood loss (EBL) was reported in 11 studies, operation time was reported in 12 studies, and follow-up duration was reported in all 15 studies. Clinical outcomes, such as pain and motor improvement were reported in 13 studies, sensory improvement was reported in 11 studies, changes in bowel function were reported in 5 studies, bladder function improvement and total complications were reported in all studies. The visual analogue scale (VAS) was employed in 3 studies, Oswestry Disability Index (ODI) was reported only in 1 study, and the Japanese Orthopaedic Association (JOA) score was reported in 3 studies.

**Table 2:** Demographic and characteristics of the included studies.

Study (Author, Year)	Country	Study Design	Surgical Technique	Sample Size (M/F)	Mean Age (Years)	Prior detethering surgery, *n* (%)	Operated Spine Level (s)	Mean Amount of Shortening (mm)	Presence of deformity (%)	Mean Follow-up (months)
Hou et al., (9)	China	Retrospective Cohort	HSAD	15 (7/8)	35.10	7 (47)	N/R	17.20	N/R	21.50
Huang et al., (10)	China	Retrospective Cohort	VCR	21 (7/14)	15.40	1 (5)	T4 (1), T6 (1), T7 (1), T8 (4), T9 (6), T9 + T12 (1), T10 (4), T11 (3)	24.40	21 (100)	45.20
Ide et al., (11)	Japan	Retrospective Cohort	VCR	7 (2/5)	40.57	2 (28.5)	L1 (7)	16.00	N/R	56.57
Kokubun et al., (12)	Japan	Retrospective Cohort	VCR	8 (6/2)	31.00	1 (12.5)	T12 (2), L1 (6)	21.00	N/R	74.40
McVeigh et al., (13)	USA	Retrospective Cohort	VCR	30 (16/14)	15.70	27 (90)	T10 (4), T11 (2), T12 (16), L1 (8)	N/R	20 (66.7)	27.30
Miyakoshi et al., (14)	Japan	Case Series	VCR	3 (2/3)	20.67	0	L1 (3)	N/R	0	48.00
Nakashima et al., (15)	Japan	Retrospective Cohort	VCR	3 (2/3)	32.70	2 (66.7)	N/R	23.30	N/R	55.20
Shi et al., (16)	China	Retrospective Cohort	VCR	6 (3/3)	35.30	0	N/R	17.00	N/R	32.00
Theodore et al., (17)	USA	Retrospective Cohort	VCR	20 (5/15)	36.25	20 (100)	T12 (16), L1 (4)	23.40	1 (5)	23.30
Wang et al., (18)	China	Retrospective Cohort	HSAD	15 (9/6)	38.10	3 (20)	N/R	17.20	N/R	21.50
Wang et al., (20)	China	Retrospective Cohort	HSAD	20 (7/13)	13.40	20 (100)	L1-S1 (8), L2-S1 (2), L1-L5 (2), L2-L5 (6), others^a^	N/R	N/R	31.20
Wang et al., (19)	China	Retrospective Cohort	HSAD	64 (28/36)	35.70	6 (9.4)	L1-S1 (24), L2-S1 (5), L1-L5 (22), L2-L5 (3), L3-S1 (4), T12-L5 (3), L1-L4 (3)	N/R	N/R	48.40
Xu et al., (21)	China	Retrospective Cohort	HSAD	7 (3/4)	15.70	4 (57)	T12-L3 (1), L1-L5 (3), L1-LS1 (3)	N/R	7 (100)	28.00
Xu et al., (22)	China	Retrospective Cohort	HSAD	24 (10/14)	27.00	7 (29)	N/R	N/R	2 (8.3)	46.00
Zhang et al., (23)	USA	Retrospective Cohort	VCR	8 (4/4)	47.50	7 (87.5)	T10 (2), T11 (3), T12 (3)	17.20	N/R	25.05
Overall				251 (111/140)	28.37			25.1		36.95

F, Female; M, Male; HSAD, Homogeneous spinal-shortening axial decompression; USA, United States of America; VCR, Vertebral column resection; N/R, Not reported.

a not further specified in the study.

### Demographics and baseline characteristics

The included studies presented a diverse cohort of patients diagnosed with tethered cord syndrome (TCS) with a mean age of approximately 28 years. Across the literature, there was a slight predominance of female patients (55.8%), though gender distribution varied between studies. The etiological spectrum of TCS encompassed a variety of pathologies, including spinal lipoma (*n* = 39), lipomyelomeningocele (*n* = 59), spina bifida (*n* = 36), lipomeningocele (*n* = 32), myelomeningocele (*n* = 30), and syringomyelia (*n* = 15), each contributing uniquely to the pathophysiological tethering of the spinal cord. All of the 15 studies reported the follow-up period of their participants, with a mean follow-up period of 36.95 ± 6.83 months. The mean follow-up period between HSAD and VCR was comparable at 32.71 and 40.46, respectively. History of prior untethering was reported in 14 studies, with Theodore, et al. and Wang et al. each studied VCR and HSAD outcomes specifically in patients with previous untethering. No significant age difference between patients in HSAD and VCR group when subgroup analysis was performed.

### Preoperative clinical data

Preoperative clinical presentations were heterogeneous but consistently featured three major symptom domains: pain (*n* = 108), motor weakness (*n* = 155), and bladder (*n* = 197) or bowel dysfunction (*n* = 57). Pain, frequently localized to the lower back or radiating to the lower extremities, was one of the most commonly reported symptoms. Motor deficits, typically manifesting as weakness in the lower limbs or gait disturbances, were also prevalent. Bladder and bowel dysfunction was another significant and debilitating symptom observed across patient groups.

### Intra-operative findings

**Figure 2:**
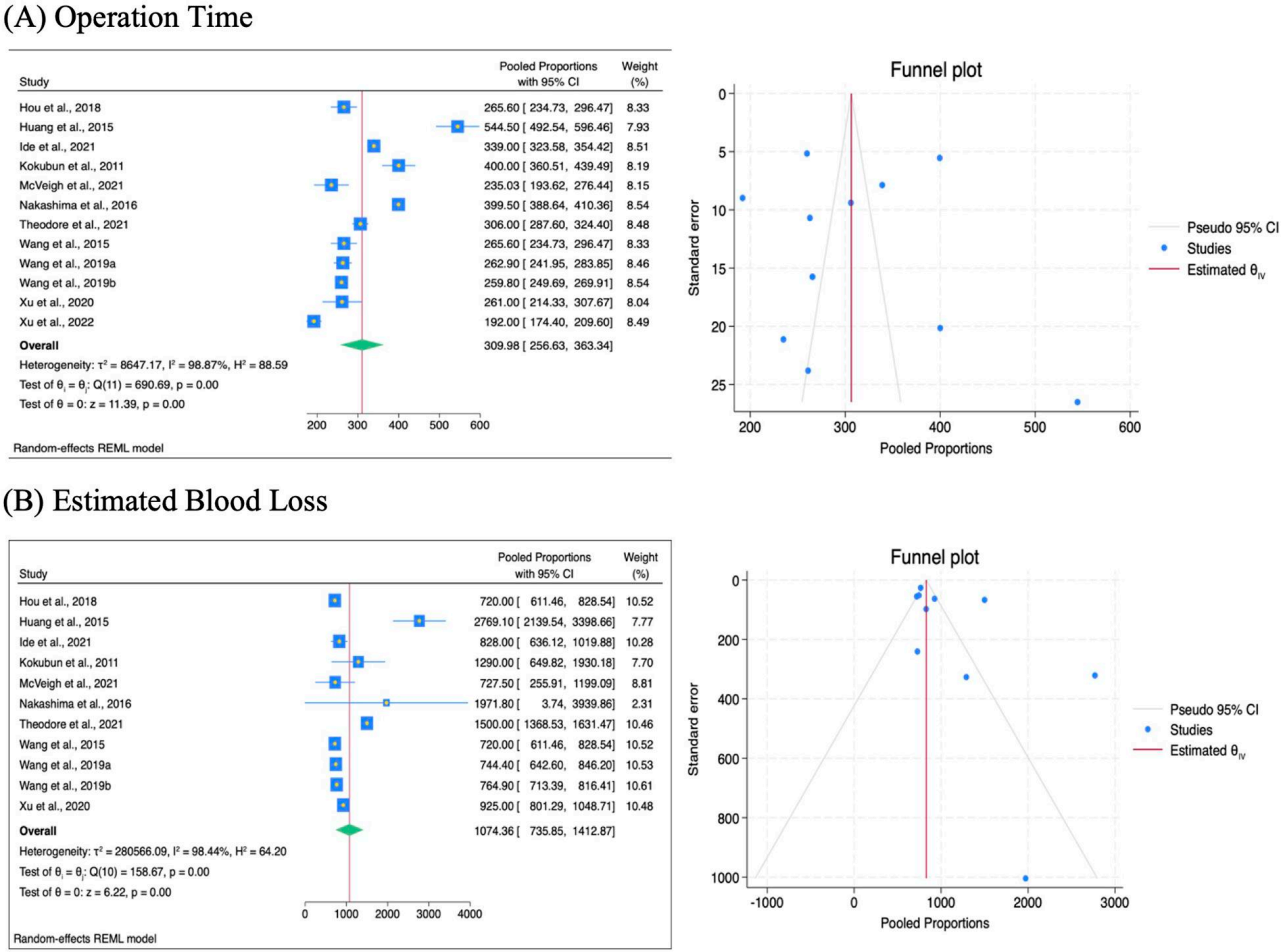
Intra operative characteristics and the relevant funnel plots. **(A)** Operation time, measured in minutes. **(B)** Estimated blood loss (EBL), measured in mL.

Operation time was reported in 12 out of 15 studies, where 3 studies on VCR had not included the operation time. Figure 2 presents the details of the intra-operative findings for the included studies. We found the overall operation time for VCR and HSAD had a mean of 309.98 ± 53.35 min. Our analysis showed that HSAD had a significantly shorter operation time at 249.69 ± 25.43 min, compared to VCR at 369.66 ± 82.3 min (*p* = 0.01). The mean estimated blood loss (EBL) for both procedures was 1,074.36 mL, while subgroup analysis showed a statistically significant smaller EBL for HSAD (768.99 mL vs. 1,435.78 mL, *p* = 0.04). Table 3 presents the result for the subgroup analysis for intra-operative findings.

**Table 3:** Subgroup analysis based on technique, follow-up duration, country, and population.

Parameter	Technique			Follow-up Duration			Country				Population			
	HSAD	VCR	*p*-value of differences	<36 months	>36 months	*p*-value of differences	China	Japan	USA	*p*-value of differences	Adult	Mixed	Pediatric	*p*-value of differences
Baseline characteristics														
Age (years ± SD)	26.99 ± 8.77	29.51 ± 7.92	0.68	30.32 ± 8.52	26.04 ± 7.66	0.46	26.02 ± 7.54	29.45 ± 9.01	32.79 ± 18.27	0.73	39.11 ± 4.92	24.44 ± 6.74	13.40 ± 1.57	<0.001^a^
Operative variables														
Operation time (mins ± SD)	249.69 ± 25.43	369.66 ± 82.3	0.01^a^	254.73 ± 32.32	366.20 ± 83.92	0.02^a^	291.56 ± 82.38	378.1 ± 41.17	273.04 ± 69.37	0.02^a^	328.63 ± 55.24	326.67 ± 95.97	262.9 ± 20.95	0.00
EBL (mL ± SD)	768.99 ± 58.05	1,435.78 ± 635.25	0.04^a^	891.01 ± 316.47	1,318.17 ± 645.9	0.24	1,060.24 ± 578.98	1,015.71 ± 439.3	1,148.31 ± 754	0.96	1,062.29 ± 463.64	1,158.66 ± 594.91	744.40 ± 101.8	0.18
Clinical Outcome														
Pain improvement (%; 95%CI)	92; 76–98	85; 74–91	0.31	90; 81–95	76; 51–90	0.12	92; 78–97	73; 44–90	87; 74–94	0.25	85; 70;94	85; 72–93	98; 71–100	0.41
Motor improvement (%; 95%CI)	77; 43–94	71; 58–81	0.67	74; 56–87	69; 44–87	0.36	77; 54–91	70; 25–94	70; 53–82	0.85	84; 66–93	68; 54–80	N/A	0.07
Sensory improvement (%; 95%CI)	66; 12–96	75; 51–89	0.76	79; 59–91	68; 33–90	0.52	76; 42–93	68; 25–93	81; 51–94	0.84	75; 42–92	74; 43–91	N/A	0.97
Bladder improvement (%; 95%CI)	82; 69–90	51; 39–63	<0.001^a^	63; 49–75	73; 43–90	0.54	80; 66–89	49; 15–84	51; 38–64	0.01^a^	54; 38–68	72; 47–88	82; 57–94	0.1
Bowel improvement (%; 95%CI)	67; 41–85	54; 39–68	0.4	52; 37–67	68; 45–85	0.26	68; 43–85	75; 11–99	52; 37–67	0.51	57; 31–80	57; 43–71	N/A	1.00
Complication Rate (%; 95%CI)	7; 4–13	17; 9–31	0.05	11; 6–18	14; 5–33	0.59	10; 4–22	13; 4–37	14; 7–26	0.81	15; 7–29	12; 6–23	2; 0–29	0.41

a Statistically significant *p*-value.

### Clinical outcomes and complications

The outcomes assessed across these studies included pain improvement, motor/sensory function improvement, and changes in bowel/bladder incontinence. Pain measurement with Visual Analog Scale (VAS) scores was reported in 3 studies (9, 19, 20). Motor function improvement was reported in 13 studies, 4 HSAD studies and 9 VCR studies. While sensory function improvement was reported only in 11 studies, 3 of them coming from HSAD studies (9–12, 14–18, 22, 23). Bowel incontinence was recorded and reported in 5 studies, only 1 of them was on HSAD (10, 11, 13, 17, 19). Bladder function was measured and compared in all studies (9–23). Overall, the studies demonstrated that both SCS procedures result in good clinical outcomes. Subgroup analyses were also performed for the clinical outcomes of SCS, which is represented in Table 3. Motor and sensory function improvement were assessed during follow-up through physical examination carried out by the surgeon in the outpatient clinic. Bowel function improvement was assessed from history taking and signs of incontinence, the studies. Bladder function was assessed mainly from history and thorough physical examinations, select studies utilized urodynamic studies to measure urodynamic parameters (9, 13, 18–22).

**Figure 3:**
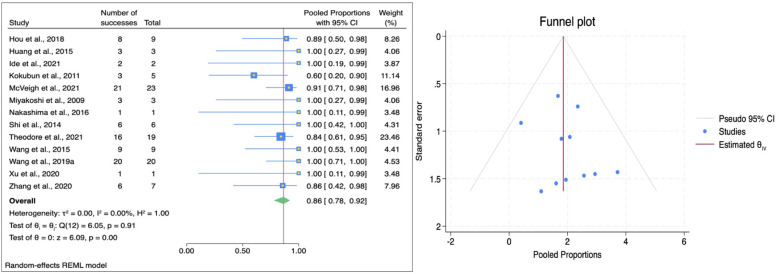
Forest plot (left) illustrating the comparison of pain improvement following HSAD and VCR in TCS; funnel plot (right) analysis of studies included for pain improvement.

The pooled effect size (ES) for pain improvement across all included studies was 0.86 (95% CI: 0.78–0.92; Figure 3), suggesting a very high likelihood of postoperative pain relief following spinal shortening procedures. The absence of statistical heterogeneity (*I*^2^ = 0.00%, *p* = 0.91) indicates that the effect was consistent across different studies and populations. When stratified by technique, HSAD yielded a pooled ES of 0.92 (95% CI: 0.76–0.98), indicating near-universal pain improvement among patients treated with this method. In contrast, VCR demonstrated a slightly lower pooled ES of 0.85 (95% CI: 0.74–0.91). However, significant statistical difference was not reached from subgroup analysis (*p* = 0.31), as seen in Table 3. Rate of pain improvement reported in studies with mean follow-up period less than 3 years were calculated around 90% (95%CI: 81–95), while studies with follow-up period more than 3 years yielded 76% (95%CI: 19–86). However, this difference is not statistically significant. Pain improvement was also similar across different population (85%; 95%CI: 70%–94% vs. 85%; 95%CI: 72%–93% vs. 98%; 95%CI: 71%–100%). Egger's test for pain improvement yielded an intercept (*β*₁) of 0.35 (SE = 0.84, *z* = 0.42, *p* = 0.677), suggesting no publication bias. Trim-and-fill procedure produced identical pooled proportion estimate for observed and adjusted proportion (1.857; 95%CI: 1.259–2.454).

**Figure 4:**
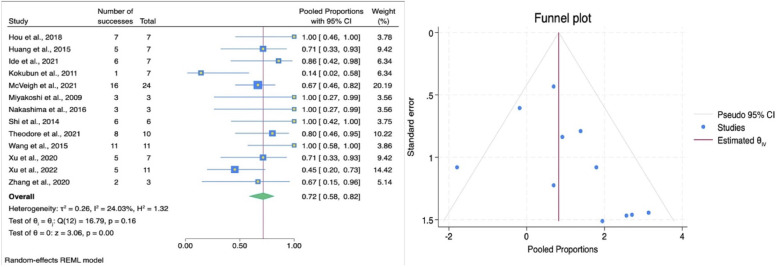
Forest plot (left) illustrating the comparison of motor function improvement following HSAD and VCR in TCS; funnel plot (right) analysis of studies included for motor function improvement.

Motor function improvement analysis showed an overall pooled ES of 0.72 (95% CI: 0.58–0.82; Figure 4), indicating a generally favorable outcome for motor recovery postoperatively. The analysis revealed a heterogeneity of 24.03%, suggesting low variability among studies. When stratified by technique, HSAD demonstrated a pooled ES of 0.77 (95% CI: 0.43–0.94) and VCR showed a slightly lower ES of 0.71 (95% CI: 0.58–0.81). These results suggest that both procedures are capable of improving motor function with no significant statistical difference between them (Table 3). Motor improvement was higher in studies with follow up duration less than 3 years (74% vs. 69%), however, significant statistical difference was not reached. No significant statistical difference was reached after subgroup analyses performing for other covariate. Assessment for publication bias was conducted using Egger's test and trim-and-fill method. Egger's test showed an intercept of 1.52 (SE = 0.80, *z* = 1.90, *p* = 0.0575), suggesting there was no statistically significant evidence of small-study effects. The trim-and-fill analysis yielded a pooled proportion of 0.824 and adjusted pooled proportion of 0.824. These findings indicate that the result is stable and not affected by publication bias.

**Figure 5:**
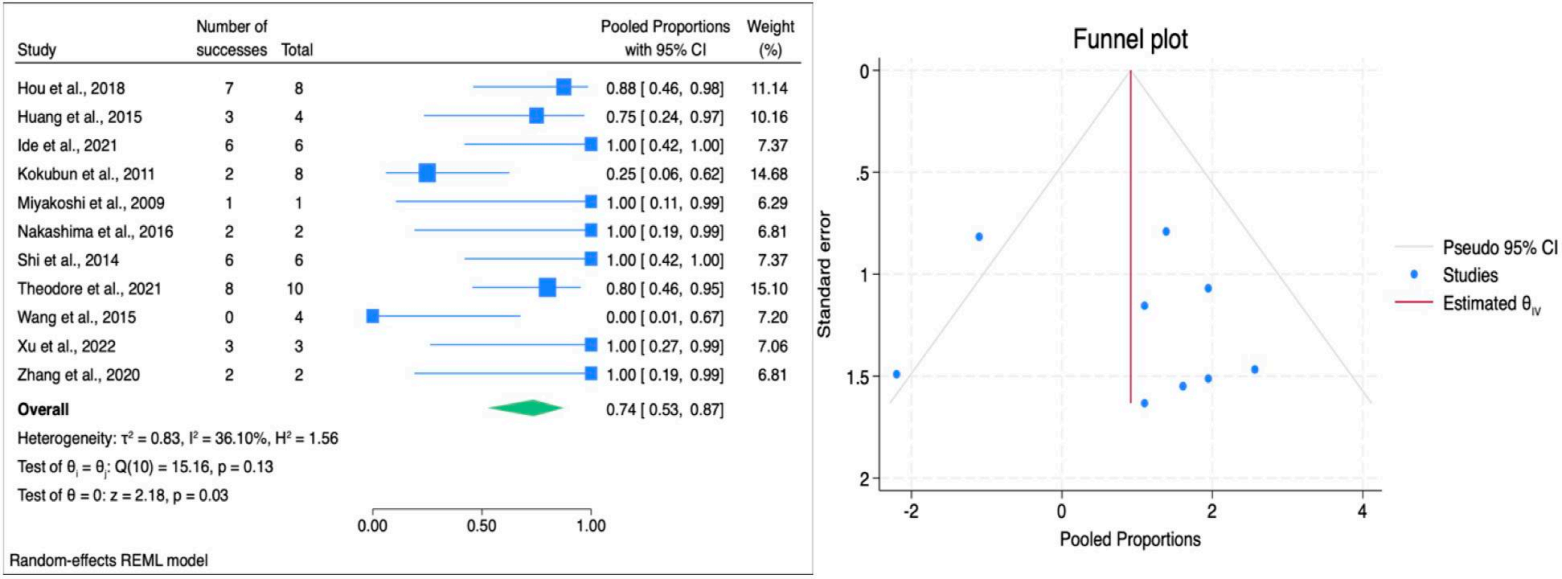
Forest plot (left) illustrating the comparison of sensory function improvement following HSAD and VCR in TCS; funnel plot (right) analysis of studies included for sensory function improvement.

The overall pooled ES for sensory function improvement was 0.74 (95% CI: 0.53–0.87; Figure 5), signifying a high rate of sensory improvement postoperatively. The overall heterogeneity was moderate (*I*^2^ = 36.1%, *p* = 0.13), indicating acceptable variability between studies. Subgroup analysis revealed that a slightly higher ES for HSAD, 0.66 (95% CI: 0.12–0.96), while VCR demonstrated a pooled ES of 0.75 (95% CI: 0.51–0.89). There was no significant difference between groups (*p* = 0.76), suggesting both procedures have similar efficacy in improving sensory function (Table 3). Egger's test for sensory improvement was performed and yielded a β₁ of 1.26 (SE = 1.49, *z* = 0.85, *p* = 0.397), indicating no significant small-study effect. Trim-and-fill analysis produced identical observed and adjusted pooled proportion. Hence, despite a modest overall heterogeneity, the pooled ES is not significantly influenced by publication bias.

**Figure 6:**
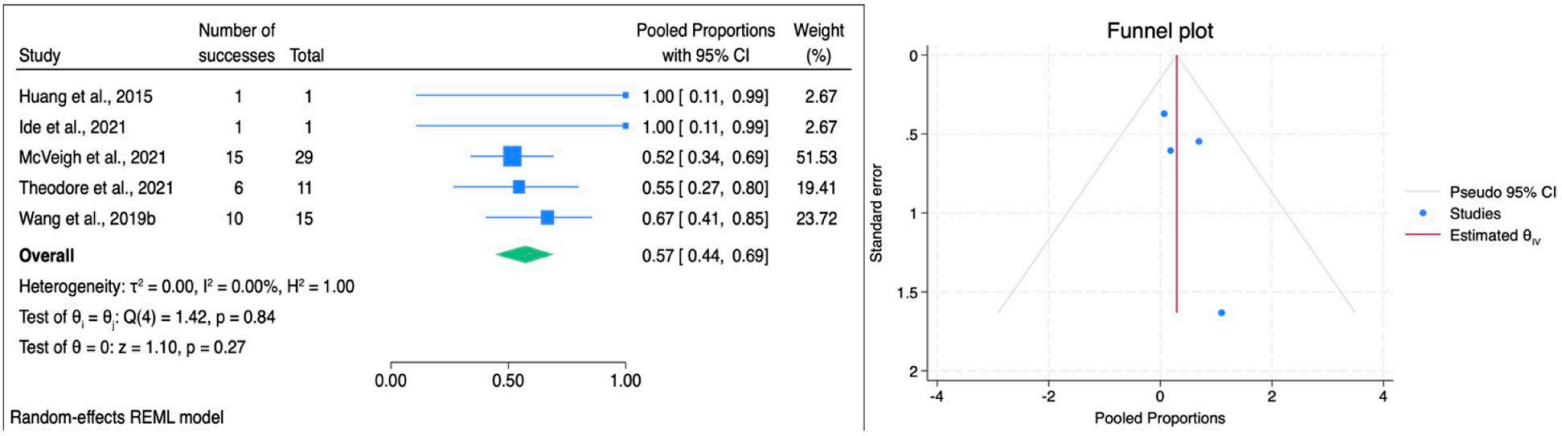
Forest plot (left) illustrating the comparison of bowel function improvement following HSAD and VCR in TCS; funnel plot (right) analysis of studies included for bowel function improvement.

The impact of HSAD and VCR on bowel function recovery in patients with TCS was demonstrated by a pooled ES of 0.57 (95% CI: 0.44–0.69; Figure 6), suggesting that approximately 57% of patients experienced improvement in bowel function following SCS procedure. Importantly, no significant heterogeneity was observed (*I*^2^ = 0.00%, *p* = 0.72), indicating a consistent effect across the studies. Subgroup analysis showed that HSAD (based on a single study by Wang et al., 2019) had an ES of 0.67 (95% CI: 0.41–0.85), while the VCR group (4 studies) showed a slightly lower pooled ES of 0.54 (95% CI: 0.39–0.68). There was no statistically significant difference between the two techniques (*p* = 0.4), implying comparable effectiveness in improving bowel function (Table 3). Bowel function improvement was observed to be consistent based on follow-up duration, country, and also patient population. Publication bias was assessed with Egger's test, which yielded an intercept of 0.83 (SE = 0.95, *z* = 0.88, *p* = 0.38). Trim-and-fill analysis produced identical pooled proportion after adjustment. These findings support that the estimate is stable and not influenced by publication bias.

**Figure 7:**
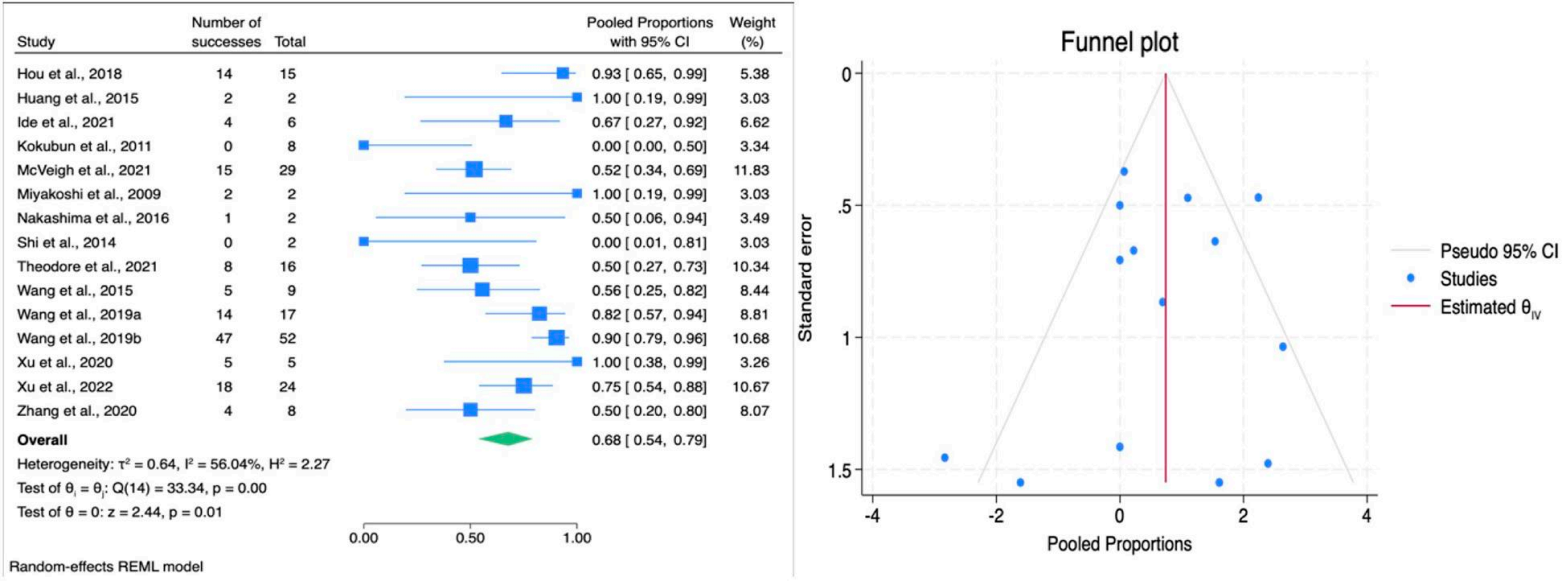
Forest plot (left) illustrating the comparison of bladder function improvement following HSAD and VCR in TCS; funnel plot (right) analysis of studies included for bladder function improvement.

Our analysis demonstrated an overall pooled ES of 0.68 (95% CI: 0.54–0.79; Figure 7) in bladder function improvement. Indicating that approximately 68% of patients experienced some degree of bladder function improvement. However, heterogeneity test resulted an estimate of 56.04%. Subgroup analysis demonstrated a statistically significant difference between techniques (*p* = 0.001). HSAD showed a substantially greater improvement (ES = 0.82, 95% CI: 0.69–0.9) compared to VCR (ES = 0.51, 95% CI: 0.39–0.63) as seen on Table 3. Moreover, studies performed in China produced higher rate of bladder function improvement compared to Japan and USA (80% vs. 49% vs. 51%; *p* = 0.01). Egger's test suggested no significant small-study effect (β_1_ = −.037, SE = 0.79, *z* = −0.47, *p* = 0.638) and a symmetrical funnel plot, hence the summary effect estimate for bladder function improvement is stable and unaffected by publication bias.

**Figure 8:**
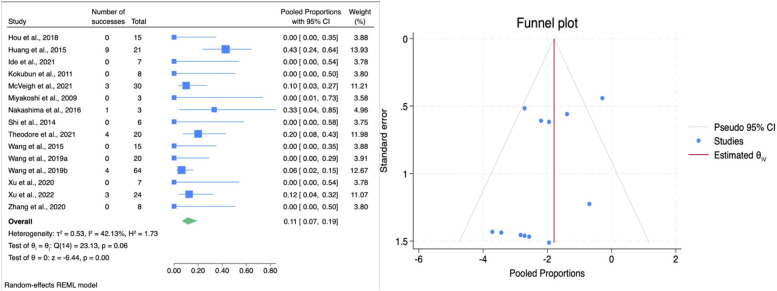
Forest plot (left) illustrating the comparison of complications following HSAD and VCR in TCS; funnel plot (right) analysis of studies included for complications.

The overall complication rate across all studies was 11% (95% CI: 7%–19%), indicating that complications were relatively rare across both surgical approaches (Figure 8). Subgroup analysis revealed no statistically significant difference in complication between HSAD and VCR with a trend of lower complication rate toward HSAD as suggested by the borderline statistical significance (*p* = 0.05). HSAD demonstrated a pooled complication rate of 7% (95% CI: 4–13, *p* = 0.05; Table 3) with no observed heterogeneity. In contrast, the VCR subgroup had a higher complication rate of 0.17 (95% CI: 0.09–0.31) and showed moderate heterogeneity (*I*^2^ = 59.80%, *p* = 0.06), indicating greater variability in outcomes among the included studies. Most common complications reported were surgical site infection and transient neurological deficits with 5 cases reported each. Cases of transient neurologic deficits included deterioration in sensory/motor function following surgery which fully recovered by the next follow-up. Permanent deteriorating neurologic deficits after surgery was reported in 4 cases (17, 20, 22). There were 4 cases of massive blood loss (>1,500 mL) during surgery reported in 3 different studies (10, 13, 15). Other complications include urinary tract infection (2 cases), CSF leakage (1 case), unintended durotomy (2 cases), and instrumentation failure (1 case). Publication bias for complication was carried out using Egger's regression test and trim-and-fill procedure. Egger's test produced a borderline result (β_1_ = −.130, SE = 0.68, *z* = −1.91, *p* = 0.056), suggesting weak evidence of asymmetry. Trim-and-fill procedure imputed 5 potentially missing studies on the right, resulting in a slightly attenuated but still significant pooled estimate (observed = −2.043, 95%CI = −2.665 to −1.422; adjusted = −1.748, 95%CI = −2.301 to −1.194). These results suggest a minor small-study effect, however the direction and statistical significance of the pooled estimates remain unchanged, confirming the robustness of the analysis.

Meta-regression analyses were conducted to investigate the potential effect of study-level variables (prior untethering, gender, age, EBL, and operation time) on the pooled ES for improvement in pain, motor and sensory function, and bladder/bowel function (Table 4). Our analysis shows that pain improvement has a constant term of 0.03 and despite a considerably high heterogeneity (*R*^2^ = 87%), none of the moderators significantly affecting the outcome. These findings indicated a significant pain improvement following SCS procedures can be expected independent of covariates. Motor improvement produced a moderate heterogeneity (*I*^2^ = 62.5%), but no study-level variable showed a significant relationship with ES. Sensory improvement also exhibited a substantial heterogeneity (*I*^2^ = 82.8%) with no moderators significantly affecting the pooled ES. Notably, bladder function improvement had a moderate heterogeneity of 52.6% and a trend toward lower improvement in patients with prior untethering was noted (β = −0.108, *p* = 0.08). Fortunately, the intercept remained significant (*p* = 0.02), supporting an overall improvement effect across studies. It is suggested that while heterogeneity varied, the included study-level covariate did not significantly account for differences in outcome and the pooled estimates remained robust statistically.

**Table 4:** Results of meta-regression analysis for clinical outcomes following SCS.

Outcome	*N* (studies)	τ^2^	*I*^2^ (%)	*R*^2^ (%)	Wald *χ*^2^ (df)	Prior untethering (p)	Male (*p*)	Age (*p*)	Estimated blood Loss (*p*)	Operation time (*p*)	Constant (*p*)
Pain Improvement	10	6.6 × 10⁻⁸	0	87.05	2.77 (5)	0.076 (0.35)	−0.094 (0.55)	−0.037 (0.44)	−0.0010 (0.49)	0.0057 (0.66)	1.305 (0.03)^a^
Motor Improvement	9	2.126	62	0.49	4.68 (5)	−0.132 (0.51)	0.436 (0.26)	0.196 (0.07)	0.0042 (0.20)	−0.0266 (0.33)	1.554 (0.21)
Sensory Improvement	7	4.67	82.8	0	0.41 (5)	0.002 (0.99)	−0.245 (0.84)	−0.008 (0.99)	0.0005 (0.95)	−0.0074 (0.86)	0.950 (0.64)
Bladder Improvement	11	0.87	52.6	5.72	4.86 (5)	−0.108 (0.08)	0.016 (0.77)	−0.031 (0.45)	0.0009 (0.52)	−0.0170 (0.17)	1.208 (0.02)^a^

a Statistically significant *p*-value.

## Discussion

This study was aimed to compare the efficacy and safety outcome of HSAD and VCR in patients with TCS. The current analysis aligns with prior observational reports and case series that have supported the use of spinal column shortening techniques in TCS. Complex TCS cases are at a higher risk of retethering following a traditional untethering, hence.

SCS is thought as the alternative in relieving the spinal cord tension while also preventing recurrence by reducing the spinal column height. A study reported a superior efficacy and safety of SCS compared to untethering surgery in patients with recurrent TCS (23). VCR was one of the first SCS procedures carried out in TCS patients. It produced great clinical outcomes, however there are several potential drawbacks needing to be addressed. Removing vertebral bodies or osteotomy can be quite challenging and even risk injury of the spinal cord. Moreover, sufficient reduction in spinal column height might be harder to achieve with single-level osteotomy. Lastly, spinal instability can be a real threat if the adjacent halves of the vertebral bodies do not fuse adequately, despite posterior instrumentation with rods and screws (20).

HSAD was first introduced in a study by Wang, et al., as a modification of spinal-shortening osteotomy to indirectly reduce the spinal cord tension in TCS (18). This technique involves removing the intervertebral disc tissues in several vertebral levels, followed by posterior instrumentation with rods and screws fixation accordingly. HSAD produces a significant reduction of the vertebral columns, hence indirectly relieving the tension on the tethered spinal cord (20). VCR is an already well-known surgery for correction of scoliosis. It involves spinal column osteotomy and removal of the vertebral body, and is typically performed through a posterior-only approach. Posterior segmental instrumentation with rods and screws are performed accordingly depending on the indications (26).

The analysis demonstrated that both HSAD and VCR were effective in improving pain in TCS patients. However, there was a slightly better outcome with HSAD compared to VCR. This could be explained by the relatively less invasive nature of HSAD which does not involve extensive vertebral resection. On the other hand, HSAD produces a more uniform shortening of the spinal column reducing neural inflammation perioperatively. Patient selection will play an important role for each technique. VCR might be more suitable in TCS patients with accompanying bony deformities, such as scoliosis, where VCR can also correct the deformity.

Improvement in motor and sensory function was demonstrated in a similar fashion by both techniques with no significant statistical difference. Notable improvements in bowel and bladder function were also reported by both techniques. Neurological deficit in TCS is resulted by the straining of the spinal cord, hence, adequate reduction in the spinal column height will remove the strain and hopefully allow recovery of the neurological function. Reducing 2–2.5 cm of the spinal column height provides equal removal of the spinal cord tension to a surgical untethering technique, with at least 90% of the neural components are successfully untethered (27, 28). Our analysis showed that motor and sensory function improvement were observed across all studies with a rate of 72% and 68%, respectively, as presented in Figures 4, 5.

The safety profile, while generally acceptable, revealed some notable complications. The most commonly reported adverse events included cerebrospinal fluid (CSF) leaks, surgical site infections, and transient neurological deficits. However, permanent neurological injuries were rare, and most complications were manageable with appropriate perioperative care. These findings suggest that, although technically demanding, both VCR and HSAD can be performed safely in experienced hands with careful patient selection. The wide confidence intervals in some VCR studies further reflect variability in complication definitions and surgical expertise, underscoring the importance of surgeon experience and procedural standardization in determining outcomes.

One of the risks following untethering surgery is recurrence of spinal cord tethering and has been reported in several studies. One study reported a retethering rate of 7.5% with a median time to retethering of 4,3 years, where some cases were asymptomatic initially (29). Another study reported retethering rate was significantly higher in complex cases compared to simple case, 33.6% and 6.6%, respectively, within 30.5 months following untethering surgery (30). In this review, with an average follow-up period of 36.95 months, no recurrence of the symptoms was reported in TCS patients following a SCS procedure.

The comparative analysis suggests that both HSAD and VCR are equally effective in improving pain and neurological functions, including motor, sensory, bowel, and bladder outcomes in patients with TCS. However, HSAD demonstrated significantly better improvement in bladder function compared to VCR. Clinical improvement was consistently seen across all studies with no significant difference between studies regardless of the follow-up period, suggesting clinical improvements following VCR and HSAD are stable even in long-term follow-up with no reported recurrence. Complication rate was observed to be lower in HSAD compared to VCR with a borderline statistical significance (*p* = 0.05). Meta-regression analyses demonstrated that study-level variables did not have significant influence on clinical outcome. Importantly, studies including patients with prior untethering surgery or complex deformity for both techniques achieved favorable functional outcomes without evidence of publication bias. These results reinforce the clinical reliability of SCS, especially HSAD and VCR, as an alternative to surgical untethering in specific cases with comorbidities or recurrent TCS.

### Limitations

Several limitations of this study must be acknowledged. First, the majority of included studies were retrospective cohort studies, which are inherently prone to selection bias, reporting bias, and lack of randomization. The variability in surgical techniques, outcome definitions, and follow-up durations across studies further limits the generalizability of the findings. Inadequate data on spinal shortening from the studies rendered analysis to be difficult. In particular, studies on VCR were often limited by smaller sample sizes and higher variability in complication rates, which may reflect differing experience levels or institutional practices rather than the technique itself. Analysis of the level of the spinal cord attachment was also not performed in this study due to inadequate data. Furthermore, publication bias could not be ruled out, as studies with negative or inconclusive results are less likely to be published. Additionally, the lack of standardized reporting on functional outcomes across studies complicates interpretation and may inflate effect estimates.

### Recommendations

To strengthen the evidence base, randomized controlled trials (RCTs) comparing HSAD and VCR in well-defined TCS populations are urgently needed. Such studies should aim to balance patient demographics, disease severity, and surgical indications to minimize confounding. While ethical challenges may exist in conducting RCTs for surgical interventions, prospective multicenter trials or registry-based comparative effectiveness studies can be an option. Future studies should also perform and provide objective measurements for bladder and bowel function to build a more robust dataset. Given the chronic nature of TCS and potential late complications or retethering, long-term follow-up studies evaluating sustained functional improvements, quality of life, and recurrence rates are essential.

## Conclusions

Spinal column shortening procedures, especially HSAD and VCR, possess great benefits for TCS patients, as a primary management or revision surgery. Both HSAD and VCR demonstrated great clinical outcomes, such as improvement in pain, motor/sensory function, and also bowel/bladder function. HSAD consistently demonstrated superior characteristics across multiple domains, especially shorter operation time, lower EBL, better bladder function improvement, and lower complication rates compared to VCR. However, VCR might be more suitable in cases with certain bony abnormalities requiring correction. Further research is necessary to assess the long-term outcome and support the use of SCS procedures in TCS.

## Data Availability

The datasets presented in this study can be found in online repositories. The names of the repository/repositories and accession number(s) can be found in the article/Supplementary Material.
